# Predictors of Memory in Healthy Aging: Polyunsaturated Fatty Acid Balance and Fornix White Matter Integrity

**DOI:** 10.14336/AD.2017.0501

**Published:** 2017-07-21

**Authors:** Marta K. Zamroziewicz, Erick J. Paul, Chris E. Zwilling, Aron K. Barbey

**Affiliations:** ^1^Decision Neuroscience Laboratory, University of Illinois Urbana-Champaign, Urbana, IL, USA.; ^2^Beckman Institute for Advanced Science and Technology, University of Illinois Urbana-Champaign, Urbana, IL, USA.; ^3^Neuroscience Program, University of Illinois Urbana-Champaign, Urbana, IL, USA.; ^4^Department of Psychology, University of Illinois Urbana-Champaign, Urbana, IL, USA.; ^5^Carle Neuroscience Institute, Carle Foundation Hospital, Urbana, IL, USA.; ^6^Department of Internal Medicine, University of Illinois Urbana-Champaign, Urbana, IL, USA.; ^7^Institute for Genomic Biology, University of Illinois Urbana-Champaign, Champaign, IL, USA

**Keywords:** nutritional cognitive neuroscience, memory, polyunsaturated fatty acids, white matter integrity, healthy aging

## Abstract

Recent evidence demonstrates that age and disease-related decline in cognition depends not only upon degeneration in brain structure and function, but also on dietary intake and nutritional status. Memory, a potential preclinical marker of Alzheimer’s disease, is supported by white matter integrity in the brain and dietary patterns high in omega-3 and omega-6 polyunsaturated fatty acids. However, the extent to which memory is supported by specific omega-3 and omega-6 polyunsaturated fatty acids, and the degree to which this relationship is reliant upon microstructure of particular white matter regions is not known. This study therefore examined the cross-sectional relationship between empirically-derived patterns of omega-3 and omega-6 polyunsaturated fatty acids (represented by nutrient biomarker patterns), memory, and regional white matter microstructure in healthy, older adults. We measured thirteen plasma phospholipid omega-3 and omega-6 polyunsaturated fatty acids, memory, and regional white matter microstructure in 94 cognitively intact older adults (65 to 75 years old). A three-step mediation analysis was implemented using multivariate linear regressions, adjusted for age, gender, education, income, depression status, and body mass index. The mediation analysis revealed that a mixture of plasma phospholipid omega-3 and omega-6 polyunsaturated fatty acids is linked to memory and that white matter microstructure of the fornix fully mediates the relationship between this pattern of plasma phospholipid polyunsaturated fatty acids and memory. These results suggest that memory may be optimally supported by a balance of plasma phospholipid omega-3 and omega-6 polyunsaturated fatty acids through the preservation of fornix white matter microstructure in cognitively intact older adults. This report provides novel evidence for the benefits of plasma phospholipid omega-3 and omega-6 polyunsaturated fatty acid balance on memory and underlying white matter microstructure.

Scientific and technological innovations in medicine continue to advance our understanding of human health and disease, with recent discoveries providing insight into an essential aspect of human biology: nutrition. At the frontiers of this effort, the interdisciplinary field of *Nutritional Cognitive Neuroscience* (1) demonstrates that age and disease-related decline in cognition depends not only upon degeneration in brain structure and function, but also on dietary intake and nutritional status. Unraveling the ways in which particular nutrients and dietary components may influence specific aspects of brain structure and function to support cognition will have profound implications for understanding healthy brain aging and for treating age-related neurological disease. As the United States experiences rapid growth in the older adult population – and a corresponding increase in the medical and economic demands of treating individuals with age-related neurological disorders – effective medical and policy recommendations to promote healthy brain aging become increasingly important, providing a catalyst for research to investigate the beneficial effects of nutrition on the aging brain.

A large body of evidence demonstrates that omega-3 (n-3) polyunsaturated fatty acids (PUFAs) have protective effects on the aging brain. PUFAs are known to contribute to neuronal membrane structural integrity, control inflammation and oxidation, and promote energy metabolism (2). High PUFA intake has been linked to better performance on tasks of memory in cross-sectional (3) and longitudinal (4) studies. Importantly, age-related decline in memory precedes clinically detectable Alzheimer’s disease, and thus presents as a potential preclinical marker of Alzheimer’s disease (5). However, it is not known whether particular patterns of PUFAs differentially influence memory.

Age-related and disease-related declines in memory are traditionally thought to rely upon hippocampal gray matter structure, but cutting-edge neuroimaging techniques suggest that memory decline may be better predicted by white matter microstructure (6). Diffusion tensor imaging (DTI) is a sensitive neuroimaging technique that determines white matter microstructural integrity by measuring diffusion properties of water (7). The most widely reported measure of DTI, fractional anisotropy (FA), indicates diffusion directionality and microstructural integrity (8). Notably, high levels of n-3 PUFAs and omega-6 (n-6) PUFAs have been shown to slow age-related decline in FA across the brain (3). However, white matter microstructure does not decline in a uniform way (9), and it is unclear whether PUFA patterns differentially support microstructure in particular regions and in turn preserve memory.

Recent findings from *Nutritional Cognitive Neuroscience* further indicate that the balance between n-3 PUFAs and n-6 PUFAs has important implications for brain health (10). Gu and colleagues demonstrate that dietary patterns high in n-3 PUFAs and n-6 PUFAs may slow age-related decline in memory by preferentially promoting white matter microstructure (3). However, the extent to which particular combinations of n-3 PUFAs and n-6 PUFAs may protect against age-related decline in memory and regional white matter microstructure is unknown. Investigating this issue is critical in the continued effort to develop dietary recommendations that improve modern Western diets, which are known to have a higher ratio of n-6 PUFA to n-3 PUFA (15:1 to 20:1) than evolutionary diets (1:1 to 2:1) (11).

Therefore, the question remains: is memory dependent on specific patterns of n-3 PUFAs and n-6 PUFAs, and is this relationship reliant upon microstructure of particular white matter regions? This study investigates the influence of plasma phospholipid n-3 PUFA and plasma phospholipid n-6 PUFA patterns on memory and the extent to which regional white matter microstructure mediates this relationship in healthy, older adults.

## MATERIALS AND METHODS

### Participants

This cross-sectional study enrolled 122 healthy elderly adult patients from Carle Foundation Hospital, a local and readily available cohort of well-characterized elderly adults. No participants were cognitively impaired, as defined by a score of lower than 26 on the Mini-Mental State Examination (12). Participants with a diagnosis of mild cognitive impairment, dementia, psychiatric illness within the last three years, stroke within the past twelve months, and cancer within the last three years were excluded. Participants were also excluded for current chemotherapy or radiation, an inability to complete study activities, prior involvement in cognitive training or dietary intervention studies, and contraindications for magnetic resonance imaging (MRI). All participants were right handed with normal, or corrected to normal vision and no contraindication for MRI. Of these 122 participants, 94 subjects had a complete dataset at time of data analysis, including neuropsychological testing, MRI, and blood biomarker analysis.

### Standard protocol approval and participant consent

This study was approved by the University of Illinois Institutional Review Board and the Carle Hospital Institutional Review Board and, in accordance with the stated guidelines, all participants read and signed informed consent documents.

### Plasma phospholipid PUFA acquisition

Plasma lipids were extracted by the method of Folch, Lees and Sloane-Stanley (13). Briefly, the internal standard (25ug each of PC17:0) was added to 200ul of serum, followed by 6mL of choloroform:methanol:BHT (2:1:100 v/v/w). The protein precipitate was removed by centrifugation (2500g, 5 mins, 4°C). Then 1.5mL of 0.88% KCl was added to the supernantent, shaken vigorously and the layers were allowed to settle for 5 minutes. The upper layer was discarded and 1ml of distilled water:methanol (1:1 v/v) was added, the tube was shaken again and the layers allowed to settle for 15 minutes. The lower layer was transferred into a clean tube and evaporated to dryness under nitrogen. The phospholipid subfraction was separated by solid-phase extraction using aminopropyl columns as described by Agren, Julkunen and Penttila (14). Then the phospholipid fraction was methylated by adding 2ml of 14% BF3-MeOH and incubating at 95°C for 1 hour (15). The supernatant containing the fatty acid methyl esters (FAMEs) was dried down under nitrogen, resuspended in 100ul of hexane, transferred into amber GC vials and stored at -20°C until the time of analysis.

The phospholipid FAMEs were analyzed by a CLARUS 650 gas chromatograph (Perkin Elmer, Boston MA) equipped with a 100m x 0.25mm i.d (film thickness 0.25µm) capillary column (SP-2560, Supelco). Injector and flame ionization detector temperatures were 250°C and 260°C, respectively. Helium was used as the carrier gas (2.5mL/min) and the split ratio was 14:1. The oven temperature was programmed at 80°C, held for 16 minutes and then increased to 180°C at a rate of 5°C/minute. After 10 minutes, the temperature was increased to 192°C at a rate of 0.5°C/minute and held for 4 minutes. The final temperature was 250°C reached at a rate of 405°C/minute and held for 15 minutes. Peaks of interest were identified by comparison with authentic fatty acid standards (Nu-Chek Prep, Inc. MN) and expressed as absolute concentration (µmol/L). The plasma phospholipid fatty acids of interest were n-3 PUFAs and n-6 PUFAs, listed in Table 1.

### Nutrient biomarker pattern analysis of plasma phospholipid PUFAs

Nutrient biomarker pattern analysis was conducted in IBM SPSS statistical software, version 24 for Macintosh. Principal component analysis was used to identify nutrient biomarker patterns (NBPs) of n-3 PUFAs and n-6 PUFAs from the thirteen-plasma phospholipid PUFAs listed in Table 1. Of these, nine plasma phospholipid PUFAs (γ-linolenic acid, eicosadienoic acid, dihomo-γ-linolenic acid, docosadienoic acid, adrenic acid, α-linolenic acid, eicosapentaenoic acid, docosapentaenoic acid, and docosahexaenoic acid) were non-normally distributed, as indicated by Shapiro-Wilk test (all p-values<0.05), and therefore log-transformed to correct for skewness of variables and subsequently considered in the analysis. The appropriate rotation method was determined by examining the factor correlation matrix: varimax rotation was chosen for a correlation matrix with values less than 0.32 and direct oblimin rotation was chosen for a correlation matrix with values greater than 0.32 (16). Statistical validity of the factor analysis was confirmed via the Kaiser-Meyer-Olkin Measure of Sampling Adequacy (>0.50) (17) and Bartlett’s Test of Sphericity (*p*<0.05) (18). Outliers were identified as participants with factor score values greater than 3.0 and removed from the dataset (19). The number of NBPs to be retained was determined by a combination of eigenvalues greater than 1.0, variance accounted for by each component, and scree plot inflection point. Interpretation of each factor was based on identifying plasma phospholipid PUFAs with an absolute loading value of greater than 0.50 on a NBP (i.e., identifying the dominant plasma phospholipid PUFAs contributing to each particular NBP). Each participant received a standardized NBP score for each pattern that corresponded to a linear combination of the plasma phospholipid PUFAs.

### Neuropsychological tests

Memory was measured by the Wechsler Memory Scale - Fourth Edition (WMS-IV) Older Adult Battery (20). This assessment measured memory by way of four indices: Auditory Memory Index, Visual Memory Index, Immediate Memory Index, and Delayed Memory Index. The Auditory Memory Index indicates a participant’s ability to remember orally presented information. The Visual Memory Index indicates a participant’s ability to remember visually presented information. The Immediate Memory Index indicates a participant’s ability to recall visually and orally presented information immediately after it is presented. The Delayed Memory Index indicates a participant’s ability to recall and recognize visually- and orally-presented information after a 20 to 30-minute delay. Participants’ raw scores on each subtest were converted to normalized scaled scores and subsequently combined into indices. Z-scores for each index were calculated and then averaged to create a composite memory score (21).

### Diffusion tensor imaging of white matter microstructure

Participants were scanned on a 3.0 T Siemens Magnetom Trio MRI system (Erlangen, Germany) using a 12 channel head coil. Whole brain diffusion tensor imaging was acquired with the following parameters: FOV = 240 × 240 mm; 72 slices, slice thickness = 2 mm; TE = 98 ms; TR = 10,000 ms; in-plane resolution = 1.875 × 1.875 mm; diffusion encoding directions = 30; *b* = 0 s/mm^2^ and 1,000 s/mm^2^. Data were processed using the University of Oxford’s Center for Functional Magnetic Resonance Imaging of the Brain (FMRIB) Software Library (FSL) release 5.0 (22) diffusion toolbox (FDT) (23,24). Eddy current correction was accomplished using the eddy_correct tool and a diffusion tensor model was fit in each voxel using the DTIFIT tool, which generates fractional anisotropy (FA) values in every voxel. FA images were further processed using the FSL tract-based spatial statistics (TBSS) (25) toolbox, which projects each subjects’ FA data onto a mean white matter skeleton, representing the white-matter tracts common to all subjects.

**Table 1:** Characteristics of sample.

Demographics	*n* = 94	Regional white matter FA	(M ± SD)
Age in years (M + SD)	69 ± 3	Corpus callosum genu	0.757 ± 0.0286
Female (%)	61	Corpus callosum body	0.754 ± 0.033
Education (%)		Corpus callosum splenium	0.845 ± 0.016
High school degree	12	Fornix	0.449 ±0.010
Some college	18	Cerebral peduncle R	0.782 ± 0.019
College degree	70	Cerebral peduncle L	0.755 ± 0.022
Income (%)		Anterior internal capsule R	0.683 ± 0.023
$15,000 – $25,000	4	Anterior internal capsule L	0.659 ± 0.024
$25,000 – $50,000	15	Posterior internal capsule R	0.769 ± 0.028
$50,000 – $75,000	23	Posterior internal capsule L	0.726 ± 0.027
$75,000 – $100,000	27	Retrolenticular internal capsule R	0.715 ± 0.033
>$100,000	31	Retrolenticular internal capsule L	0.679 ± 0.027
BMI (M + SD)	26±4	Anterior corona radiata R	0.535 ± 0.032
Depression indicated (%)	6%	Anterior corona radiata L	0.523 ± 0.032
**Plasma phospholipid PUFAs**	**(M ± SD, umol/L)**	Superior corona radiata R	0.592 ± 0.029
Linoleic acid (18:2n-6)	601.2 + 149.9	Superior corona radiata L	0.558 ± 0.027
γ-linolenic acid (18:3n-6)	2.7 ± 1.6	Posterior corona radiata R	0.573 ± 0.030
Eicosadienoic acid (20:2n-6)	9.2 ± 2.6	Posterior corona radiata L	0.588 ± 0.035
Dihomo-γ-linolenic acid (20:3n-6)	70.9 ± 25.7	Posterior thalamic radiation R	0.682 ± 0.040
Arachidonic acid (20:4n-6)	295.7 ± 66.6	Posterior thalamic radiation L	0.671 ± 0.036
Docosadienoic acid (22:2n-6)	0.3 ±0.1	Sagittal stratum R	0.655 ± 0.035
Adrenic acid (22:4n-6)	10.5 ± 3.2	Sagittal stratum L	0.624 ± 0.030
α-linolenic acid (18:3n-3)	5.2 ± 2.5	External capsule R	0.567 ± 0.042
Stearidonic acid (18:4n-3)	2.3 ± 0.9	External capsule L	0.549 ± 0.031
Eicosatrienoic acid (20:3n-3)	1.2 ± 0.4	Cingulate part of cingulum R	0.631 ± 0.031
Eicosapentaenoic acid (20:5n-3)	24.7 ± 17.7	Cingulate part of cingulum L	0.669 + 0.034
Docosapentaenoic acid (22:5n-3)	22.9 + 6.9	Hippocampal part of cingulum R	0.759 + 0.032
Docosahexaenoic acid (22:6n-3)	78.6 ±32.4	Hippocampal part of cingulum L	0.719 + 0.039
**Cognition**	**(M ± SD)**	Superior longitudinal fasciculus R	0.600 ± 0.030
WMS-IV Auditory Memory Index	113 ± 13	Superior longitudinal fasciculus L	0.568 ± 0.027
WMS-IV Verbal Memory Index	112 ± 12	Superior fronto-occipital fasciculus R	0.638 ± 0.042
WMS-IV Immediate Memory Index	115 ± 12	Superior fronto-occipital fasciculus L	0.586 ± 0.042
WMS-IV Delayed Memory Index	113 ± 13	Uncinate fasciculus R	0.589 ± 0.053
Composite memory score	113 ± 11	Uncinate fasciculus L	0.561 ± 0.048
		Tapetum R	0.664 ± 0.070
		Tapetum L	0.728 ± 0.096

Abbreviations: mean (M), standard deviation (SD), body mass index (BMI), polyunsaturated fatty acid (PUFA), Wechsler Memory Scale- Fourth Edition (WMS-IV), fractional anisotropy (FA), right hemisphere (R), left hemisphere (L)

Mean FA within the white matter skeleton for specific regions of interest were calculated for each subject using the JHU ICBM DTI-81 atlas (26). The regions of interest are listed in Table 1.

### Covariates

Covariates were included according to previous association with cognitive decline (27–32). The covariates included age (continuous), gender (nominal, man/woman), education (nominal, five fixed levels), income (nominal, six fixed levels), body mass index (continuous, hereafter BMI), and depression status (nominal, yes/no). Although all participants had received a diagnosis of no depression at enrollment, the SF-36 Health Survey (33) revealed six participants with symptoms consistent with depression. Thus, in accordance with prior studies (34), this was considered in the analysis as a covariate.

### Mediation analysis

**Figure 1.**
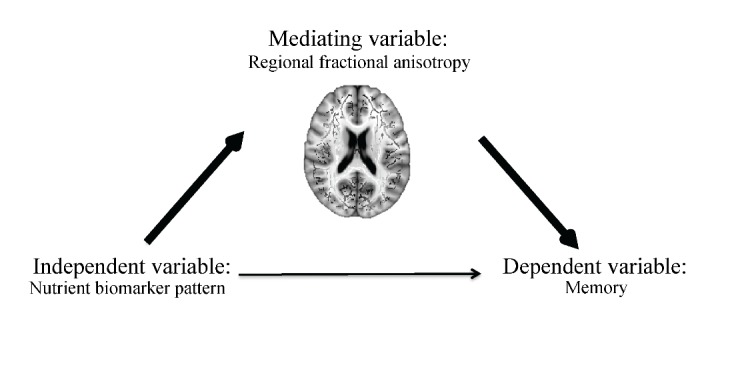
Proposed mediation model The primary requirement for mediation is a significant indirect mediation effect, defined as the effect of the independent variable (nutrient biomarker pattern) through the mediation (fractional anisotropy in white matter regions) on the dependent variable (memory).

A formal mediation analysis was conducted to model the relationship between NBPs, regional FA, and memory using a three-step framework, with the goal of evaluating whether regional FA mediated the relationship between NBPs and memory. The primary requirement for mediation is a significant indirect mediation effect (35), or the effect of the independent variable (NBPs) through the mediator (regional FA) on the dependent variable (memory) (Fig 1).

Mediation analyses were performed using the PROCESS macro designed for SPSS (36). Statistics were performed as follows:

1. In the first step, a regression model was applied to characterize the relationship between NBPs and FA of white matter regions (path a). This analysis accounted for covariates listed in *Covariates* and applied a false discovery rate (FDR) (37) correction for multiple comparisons (*q* < 0.05, one-tailed)
2. In the second step, a regression model was used to characterize the relationship between NBPs and memory (path c). This analysis accounted for covariates listed in *Covariates*.
3. In the third step, the PROCESS macro was applied to implement the bootstrapping method to estimate mediation effects. This analysis drew 1000 bootstrapped samples with replacement from the dataset to estimate a sampling distribution for the indirect and direct mediation effects, controlling for covariates listed in *Covariates*. The indirect mediation effect refers to the pathway from NBPs to regional FA to memory (path a-b). The direct mediation effect refers to the direct pathway from NBPs to memory, accounting for the effect of regional FA (path c’).

A statistically significant mediation that matches the hypothesized framework is indicated by: (i) an indirect mediation effect that does not include zero within 95% bias-corrected confidence intervals, and (ii) a direct mediation effect that does include zero within 95% bias-corrected confidence intervals (35). Results are reported using unstandardized regression coefficients (β) and statistical significance (*p*) for each individual regression relationship, and a 95% bias-corrected confidence interval (95% CI) for the direct and indirect effects of the mediation.

**Table 2:** Nutrient biomarker pattern construction: Pattern structure and variance explained^1^.

Plasma phospholipid PUFAs	NBP^2^		
	1	2	3
α-linolenic acid (18:3n-3)	0.780^*^		
Eicosadienoic acid (20:2n-6)	0.757^*^	0.328	
Eicosatrienoic acid (20:3n-3)	0.756^*^		
Linoleic acid (18:2n-6)	0.707^*^		
Docosadienoic acid (22:2n-6)	0.601^*^		
Adrenic acid (22:4n-6)		0.928^*^	
Arachidonic acid (20:4n-6)		0.767^*^	0.304
γ-linolenic acid (18:3n-6)		0.696^*^	
Dihomo-γ-linolenic acid (20:3n-6)	0.325	0.643^*^	
Stearidonic acid (18:4n-3)	0.378	0.606^*^	
Eicosapentaenoic acid (20:5n-3)			0.951^*^
Docosahexaenoic acid (22:6n-3)			0.910^*^
Docosapentaenoic acid (22:5n-3)		0.313	0.752^*^
**Percent variance explained by each NBP**	41.33	16.63	11.24
**Cumulative percent variance explained with each extraction**	41.33	57.96	69.21

Abbreviations: nutrient biomarker pattern (NBP), polyunsaturated fatty acid (PUFA)

1 Extraction method: principal component analysis; rotation method: oblimin

2 NBP interpretation was based on strongest loading coefficients within each pattern; only loadings with an absolute value > 0.3 are shown in the table

* Nutrients with absolute loadings > 0.5 that are considered as dominant nutrients contributing to the particular nutrient pattern

## RESULTS

### Participant characteristics

Participants had a mean age of 69 years and 61 percent of participants were females. All other participant characteristics are reported in Table 1.

### Nutrient biomarker patterns of plasma phospholipid PUFAs

**Figure 2.**
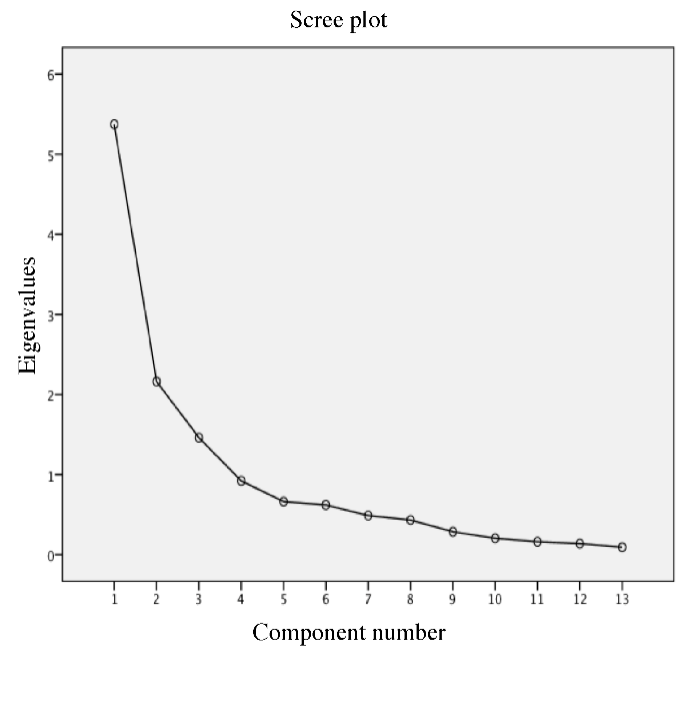
**Scree plot**: inspection of the scree plot visually indicates which nutrient biomarker patterns explain the most variability in the data. A change in curvature, or inflection point, occurred after the third component, or nutrient biomarker pattern, was extracted. Thus, three components explained most variability in the data.

Principal component analysis generated three NBPs (Table 2). The factor correlation matrix contained values greater than 0.32, therefore direct oblimin rotation was implemented. Statistical validity of the factor analysis was confirmed via the Kaiser-Meyer-Olkin Measure of Sampling Adequacy (0.762) and Bartlett’s Test of Sphericity (*p*<0.001). One outlier was removed and the principal component analysis was rerun (n=95). Three NBPs were selected for retention because (i) after the third NBP extraction with principal component analysis, 69.21 percent of the total variance was accounted for in the original set of plasma phospholipid PUFAs, and (ii) inspection of the scree plot indicated that the inflection point occurred after the third NBP (Fig 2). The dominant plasma phospholipid PUFAs contributing to each NBP were those that had an absolute loading value of greater than 0.50 on a NBP. Thus, NBP1 consisted of five plasma phospholipid PUFAs, NBP2 consisted of five plasma phospholipid PUFAs, and NBP3 consisted of three plasma phospholipid PUFAs (Table 2). Hereafter, NBP1 is described as LCPUFA (i.e., it is composed of two plasma phospholipid n-3 PUFAs and three plasma phospholipid n-6 PUFAs), NBP2 is described as n6PUFA (i.e., it is composed of four plasma phospholipid n-6 PUFAs and only one plasma phospholipid n-3 PUFA), and NBP3 is described as n3PUFA (i.e., it is composed of only plasma phospholipid n-3 PUFAs).

### Mediation results

The mediation analyses indicated that FA of one region (fornix) fully mediated the relationship between one NBP (LCPUFA) and memory. Each relationship within the mediation is described below in a stepwise fashion.

**Figure 3.**
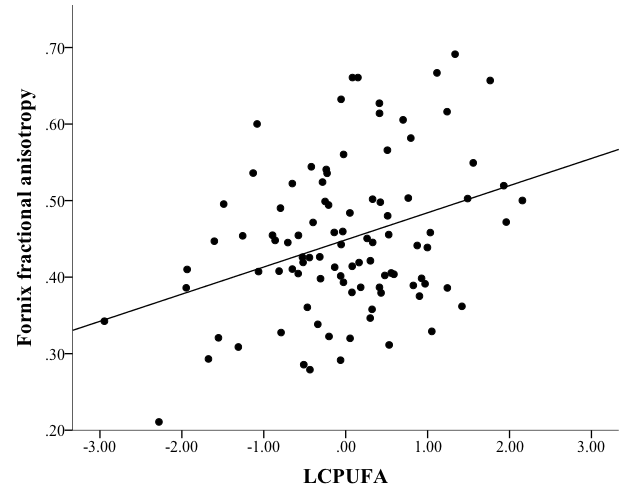
**Mediation path a**: linear regression modeling showed that nutrient biomarker pattern 1 (LCPUFA) positively and reliably associated with fornix fractional anisotropy (=0.042, *p*<0.001).

**Figure 4.**
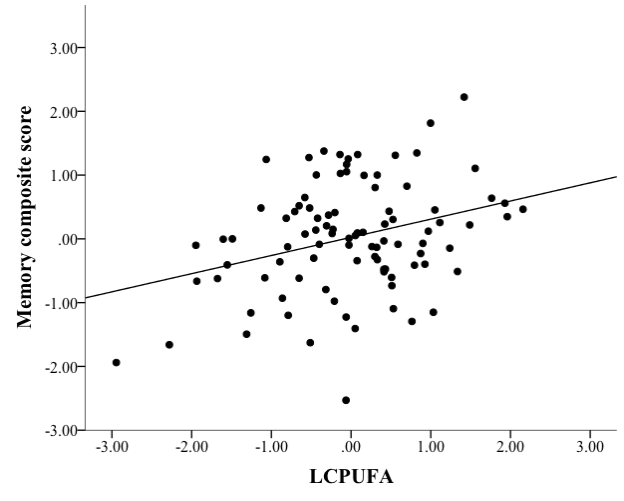
**Mediation path c**: linear regression modeling showed that nutrient biomarker pattern 1 (LCPUFA) positively and reliably associated with memory (=0.320, *p*=0.003).

**Figure 5.**
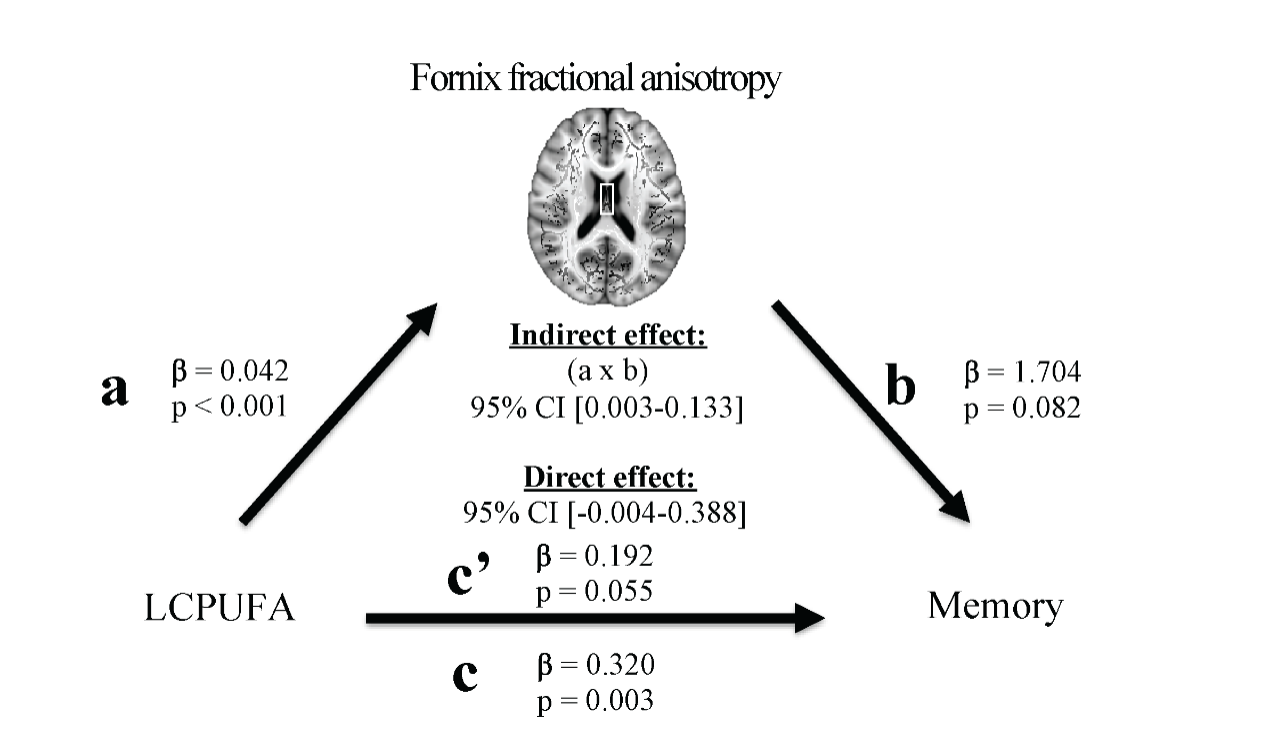
**Mediation model statistics**: nutrient biomarker pattern 1 (LCPUFA) positively associated with fractional anisotropy of the fornix (**path a**). LCPUFA positively associated with memory (**path c**). The indirect pathway of mediation (i.e., the effect of LCPUFA through fornix fractional anisotropy on memory; **path a-b**) was statistically significant. The direct pathway of mediation (i.e., the effect of LCPUFA on memory, accounting for fornix fractional anisotropy; **path c**’) was not significant. Therefore, fornix fractional anisotropy fully mediated the relationship between LCPUFA and memory.

1. Higher LCPUFA was associated with higher FA in the fornix (β=0.042, *p*<0.001; Fig 3), but no other NBP related to FA in any other region (Table 3). Therefore, the relationship between LCPUFA and fornix FA was considered in the context of the mediation model (Fig 5, path a).
2. Higher LCPUFA associated with a higher memory score (β=0.320, *p*=0.003; Fig 4), but no other NBP related to performance on memory (Table 4). Therefore, the relationship between LCPUFA and memory was considered in the context of the mediation model (Fig 5, path c).
3. The indirect pathway of mediation (the pathway from LCPUFA to fornix FA to memory) was significant (95% CI [0.003 - 0.133], Fig 5, path a-b;β=1.704, *p*=0.082; Fig 5, path b), but the direct pathway of mediation (the direct pathway from LCPUFA to memory, accounting for the effect of fornix FA) was not significant (95% CI [-0.004 - 0.388],β=0.192, *p*=0.055, Fig 5 path c’). Therefore, the mediation indicated that fornix FA fully mediated the relationship between LCPUFA and memory (Fig 5).

## DISCUSSION

This study revealed that memory is dependent upon a particular pattern of plasma phospholipid PUFAs, which comprised a mixture of plasma phospholipid n-3 PUFAs and plasma phospholipid n-6 PUFAs, and that white matter integrity of a specific white matter region, the fornix, mediates this relationship. This report provides a novel link between a combination of plasma phospholipid PUFAs, white matter microstructure of one region, and memory. The individual relationships reported within the mediation, including those between plasma phospholipid PUFAs and white matter microstructure (Fig 5, path a), between plasma phospholipid PUFAs and memory (Fig 5, path c), and between fornix white matter microstructure and memory (Fig 5, path b) are each supported by previous work and are reviewed in turn below.

The first relationship demonstrated a positive association between LCPUFA and FA of the fornix. Although previous work has not directly examined the relationship between plasma phospholipid PUFAs and white matter microstructure of the fornix, several lines of evidence support this finding. Various diets and dietary components, including the Mediterranean diet (38), PUFAs (3,34), vitamin E (3), vitamin D (39), vitamin B1 (40), and vitamin B12 (41) have been linked to white matter integrity. The fornix in particular is vulnerable to the white matter atrophy induced by Alzheimer’s pathology (42). However, loss of white matter integrity in the fornix is amenable to lifestyle factors (43), suggesting the potential for intervention with nutritional factors. This study indicates that LCPUFA is linked to a sensitive measure of fornix degeneration (44), measured by highly restricted diffusion within the fornix and represents superior myelination and a high number of myelinated nerve fibers (45).

**Table 3:** Nutrient biomarker patterns associated with regional fractional anisotropy.

Regional FA	LCPUFA	n6PUFA	n3PUFA
Corpus callosum genu	0.001(0.823)	0.001(0.693)	-0.003(0.458)
Corpus callosum body	0.007(0.105)	0.005(0.221)	-0.001(0.708)
Corpus callosum splenium	-0.001(0.727)	0.001(0.533)	-0.001(0.731)
Fornix	0.042(<0.001) ^*^	-0.008(0.426)	-0.024(0.021)
Cerebral peduncle R	<0.001(0.882)	-0.001(0.752)	-0.002(0.426)
Cerebral peduncle L	-0.002(0.427)	0.001(0.620)	-0.001(0.611)
Anterior limb of internal capsule R	<0.001(0.819)	0.004(0.103)	-0.004(0.166)
Anterior limb of internal capsule L	-0.002(0.434)	0.004(0.160)	<0.001(0.955)
Posterior limb of internal capsule R	-0.007(0.045)	0.001(0.735)	0.002(0.577)
Posterior limb of internal capsule L	-0.006(0.071)	0.001(0.647)	0.001(0.796)
Retrolenticular part of internal capsule R	-0.005(0.252)	-0.005(0.208)	0.007(0.074)
Retrolenticular part of internal capsule L	<0.001(0.965)	-0.003(0.300)	<0.001(0.953)
Anterior corona radiata R	0.004(0.306)	<0.001(0.976)	-0.006(0.101)
Anterior corona radiata L	0.004(0.280)	-0.002(0.654)	-0.003(0.437)
Superior corona radiata R	0.002(0.563)	-0.001(0.740)	<0.001(0.951)
Superior corona radiata L	<0.001(0.964)	0.001(0.721)	-0.001(0.696)
Posterior corona radiata R	0.001(0.817)	-0.002(0.646)	0.001(0.751)
Posterior corona radiata L	<0.001(0.989)	-0.002(0.607)	0.003(0.489)
Posterior thalamic radiation R	0.004(0.429)	-0.006(0.214)	-0.001(0.886)
Posterior thalamic radiation L	0.003(0.550)	-0.001(0.884)	>0.001(0.924)
Sagittal stratum R	<0.001(0.982)	-0.003(0.461)	0.002(0.665)
Sagittal stratum L	0.003(0.468)	0.002(0.643)	-0.007(0.044)
External capsule R	0.004(0.484)	0.003(0.443)	-0.011(0.017)
External capsule L	0.002(0.581)	0.002(0.609)	-0.003(0.435)
Cingulate part of cingulum R	0.008(0.051)	-0.003(0.306)	-0.004(0.264)
Cingulate part of cingulum L	0.004(0.343)	<-0.001(0.999)	-0.002(0.648)
Hippocampal part of cingulum R	-0.001(0.777)	0.001(0.882)	<0.001(0.977)
Hippocampal part of cingulum L	0.004(0.365)	-0.005(0.233)	-0.002(0.619)
Superior longitudinal fasciculus R	0.003(0.504)	-0.005(0.129)	-0.001(0.775)
Superior longitudinal fasciculus L	<0.001(0.987)	-0.001(0.800)	-0.001(0.773)
Superior fronto-occipital fasciculus R	0.003(0.559)	0.000(0.922)	-0.005(0.321)
Superior fronto-occipital fasciculus L	0.003(0.632)	-0.003(0.472)	-0.004(0.432)
Uncinate fasciculus R	0.007(0.324)	-0.001(0.846)	-0.010(0.123)
Uncinate fasciculus L	0.003(0.638)	-0.001(0.914)	-0.002(0.712)
Tapetum R	0.002(0.806)	-0.003(0.735)	0.003(0.716)
Tapetum L	-0.007(0.515)	-0.003(0.757)	-0.022(0.045)

Abbreviations: fractional anisotropy (FA), nutrient biomarker pattern 1 (LCPUFA), nutrient biomarker pattern 2 (n6PUFA), nutrient biomarker pattern 3 (n3PUFA), right (R), left (L) Model: regional FA = LCPUFA + n6PUFA + n3PUFA + age + gender + education + income + body mass index + depression status Results are presented as *β*(*p*)

* *p* < 0.05, FDR-corrected

**Table 4:** Nutrient biomarker patterns associated with memory.

NBP	Composite memory score
LCPUFA	0.320(0.003) ^*^
n6PUFA	-0.126(0.191)
n3PUFA	-0.062(0.536)

Abbreviations: nutrient biomarker pattern (NBP), nutrient biomarker pattern 1 (LCPUFA), nutrient biomarker pattern 2 (n6PUFA), nutrient biomarker pattern 3 (n3PUFA)

Model: composite memory score = LCPUFA + n6PUFA + n3PUFA + age + gender + education + income + body mass index + depression status

Results are presented as *β*(*p*)

* *p* < 0.05

The second relationship indicated a positive association between LCPUFA and memory. High PUFA intake has been linked to better performance on tasks of memory in cross-sectional (3) and longitudinal studies (4), but positive findings are not consistent (46). Most studies have investigated the effects of n-3 PUFAs on memory (10), but these findings suggest that a mixture of n-3 PUFAs and n-6 PUFAs may slow age-related decline in memory.

Third, FA of the fornix fully mediated the relationship between LCPUFA and memory. The fornix consists of bilateral white matter bundles that originate from the bilateral hippocampi, and represents a core element of the limbic circuit that is vulnerable to Alzheimer’s disease (44). Fornix microstructure has been linked to conversion from healthy aging to cognitive impairment (47) as well as memory performance in cross-sectional and longitudinal investigations (48). Given its vulnerability to Alzheimer’s disease, and its role in memory, the fornix has been proposed as a target in novel therapies for Alzheimer’s disease (44). The present findings support focusing on the fornix as a therapeutic target, and highlight the potential for nutritional interventions.

The predictive power of only one NBP, the LCPUFA pattern, suggests that particular physiological mechanisms may be important in the preservation of white matter microstructure and memory. The NBP analysis yielded three patterns of plasma phospholipid PUFAs: (i) the LCPUFA pattern consisted of both plasma phospholipid n-3 PUFAs and plasma phospholipid n-6 PUFAs that serve as precursors to long-chain PUFAs, (ii) the n6PUFA pattern primarily consisted of primarily plasma phospholipid n-6 PUFAs and one plasma phospholipid n-3 PUFA, and (iii) the n3PUFA pattern consisted of only plasma phospholipid n-3 PUFAs. All PUFAs can serve two basic functions: components of plasma membranes or precursors to prostanoids, which are vital pro- and anti-inflammatory regulatory factors (49). Importantly, n-3 PUFAs and n-6 PUFAs share the same biosynthetic enzymes, and are therefore converted to long-chain PUFAs and prostanoids in a competitive manner (50). A balance of n-3 PUFAs and n-6 PUFAs is physiologically favorable because it allows for proportional production of long-chain PUFAs for integration into plasma membranes or conversion into prostanoids (50). Therefore, the predictive power of the LCPUFA pattern suggests that it is a mixture of plasma phospholipid n-3 PUFAs and plasma phospholipid n-6 PUFAs possibly reflective of a balance of plasma phospholipid n-3 PUFAs and plasma phospholipid n-6 PUFAs, rather than the individual effects of only plasma phospholipid n-3 PUFAs or only plasma phospholipid n-6 PUFAs, that is robustly linked to memory and white matter microstructure in the aging brain. These findings imply that modern Western diets may be improved by balancing intake of dietary sources of n-3 PUFAs and n-6 PUFAs (11). Importantly, this study does not relate dietary intake of PUFAs to plasma phospholipid n-3 PUFAs and plasma phospholipid n-6 PUFAs, and future work is needed to determine how dietary intake of PUFAs may support memory and white matter microstructure in the aging brain.

The strengths of this study include: (i) the use of blood biomarkers to measure physiological status of plasma phospholipid PUFAs, (ii) the use of structural MRI to measure microstructural integrity with high spatial resolution, and (iii) the assessment of a particular aspect of cognition known to be sensitive to age-related decline and neural degeneration. Directions for future research include: (i) replication of results in a larger sample, (ii) assessment of dietary intake and its link to plasma phospholipid n-3 PUFAs and plasma phospholipid n-6 PUFAs (ii) determination of the optimal ratio of n-3 PUFAs to n-6 PUFAs, (iii) implementation of a longitudinal study to examine how changes in PUFA balance relate to changes in memory and white matter microstructure, (iv) examination of the mechanisms that support the relationship between PUFAs and fornix white matter microstructure, and (v) investigation of the relationship between PUFA balance, cognition, and white matter microstructure in other model systems, including animal models and clinical populations.

Research at the frontline of *Nutritional Cognitive Neuroscience* aims to incorporate sensitive measures of nutritional intake, brain structure and function, and cognition in an effort to demonstrate that cognitive aging is dependent not only on degeneration in brain structure and function, but also on nutritional status and dietary intake. In doing so, *Nutritional Cognitive Neuroscience* strives to bridge the gap between largely disparate literatures of aging within the fields of nutritional epidemiology and cognitive neuroscience, and offer a novel perspective on healthy brain aging. Accumulating evidence suggests that certain nutrients may slow or prevent aspects of age-related cognitive decline by influencing particular age-related changes in brain structure (1,3,51). The present finding contributes to this research program, and provides novel evidence for the benefits of n-3 PUFA to n-6 PUFA balance on memory and underlying white matter microstructure. Ultimately, this line of work can inform clinical nutritional interventions for healthy brain aging.
